# Body Mass Index and Advanced Age in Patients Undergoing Transcatheter Aortic Valve Replacement

**DOI:** 10.1016/j.jacadv.2026.103074

**Published:** 2026-07-24

**Authors:** Aryan Mehta, Saraschandra Vallabhajosyula

**Affiliations:** a Division of Cardiology, Department of Medicine, Warren Alpert Medical School of Brown University, Providence, Rhode Island, USA; b Brown University Health Cardiovascular Institute, Providence, Rhode Island, USA

**Keywords:** age, aortic stenosis, body mass index, registry, transcatheter aortic valve replacement

**Figure:**
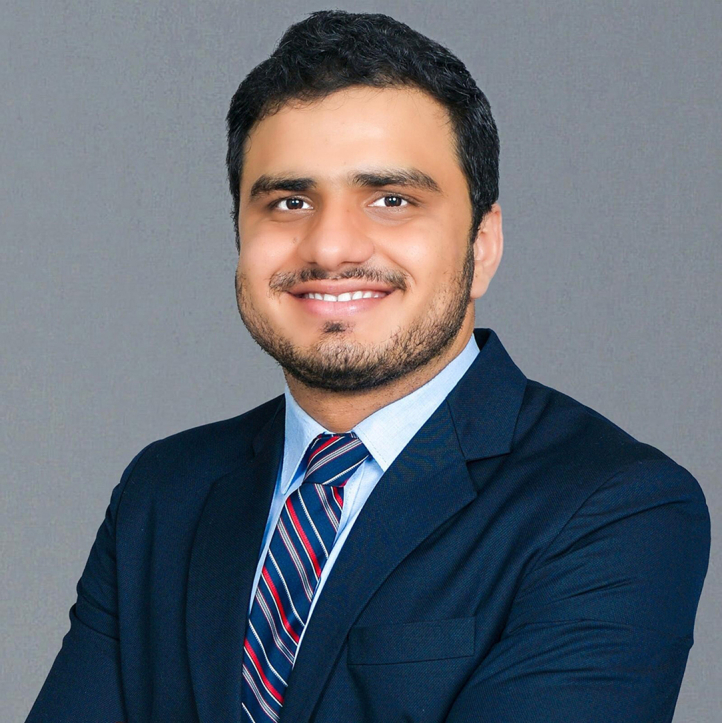

Aortic stenosis (AS) affects approximately 5% of all individuals aged 65 years or older in Western countries and remains one of the most common valvular heart diseases.^1^ Aortic valve replacement, the only definitive treatment, is a class I recommendation for symptomatic severe AS and patients with asymptomatic severe AS with a left ventricular ejection fraction of <50%, a positive stress test, and with other indications for cardiac surgery.^2^ In contemporary practice, transcatheter aortic valve replacement (TAVR) remains a highly effective treatment strategy for the treatment of severe AS, irrespective of the surgical risk.^3^ This is attributable to technical advancements in the field and partly to enhanced risk stratification by recognition and optimization of preprocedural risk factors. Obesity, which affects over 40% of adults in the United States, is a well-established risk factor for various cardiovascular conditions. The association between obesity and TAVR outcomes has previously been explored due to differences in vascular access, nutritional status, echocardiographic parameters, and respiratory status among other factors.^4^ Despite the higher prevalence of various comorbid conditions in individuals with obesity, it has often been linked to improved outcomes in several disease processes, a phenomenon commonly referred to as “obesity paradox”.^5^^,^^6^

The association between body mass index (BMI) and outcomes following TAVR has infrequently been studied, with existing literature yielding ambiguous and often conflicting results. In this issue of *JACC: Advances*, Kumar et al^7^ report the findings of their analysis titled “Clarifying the Obesity Paradox in Transcatheter Aortic Valve Replacement: Findings from the STS/ACC TVT Registry.” This retrospective analysis from a single health system performed between January 2021 and February 2023, categorized 6,639 patients by age (<65 or ≥65 years) and BMI (<25 or ≥25 kg/m^2^). Compared to their lower BMI counterparts, patients with higher BMI had a higher prevalence of baseline comorbidities, irrespective of age group. In patients ≥65 years old, the primary outcome of all-cause mortality was higher in the cohort with a BMI <25 kg/m^2^ (8.5% vs 6.3%; adjusted HR [aHR]: 1.29; 95% CI: 1.05-1.57; *P* = 0.013) when compared to the higher BMI group. In addition, the incidence of secondary outcomes, including major adverse cardiovascular and cerebrovascular events (26.8% vs 21.2%; aHR: 1.20; 95% CI: 1.08-1.34; *P* = 0.001) and readmission (21.8% vs 18.2%; aHR: 1.17; 95% CI: 1.04-1.31; *P* = 0.008) was also higher in the lower BMI cohort among patients ≥65 years of age. No such differences in primary and secondary outcomes were noted between the BMI groups among patients <65 years of age.

Even though other studies have sporadically evaluated the association between obesity and TAVR outcomes,^3^^,^^4^ the current study deconstructs this paradigm by using age as an effect modifier. They further conducted a categorical interaction analysis in which age was examined across 4 different categories. The relationship strengthened with advancing age, as demonstrated in their age-stratified and spline-based analyses. The prevalence of obesity was associated with significantly lower mortality in patients older than 75 years. This is an important distinction from prior studies, which have not explored this paradox in the context of advancing age.^3^^,^^4^ By examining effect heterogeneity by age, the authors provide a more granular characterization of the obesity paradox in this population. Another key strength of the study is the rigorous statistical analysis. The authors used inverse probability of treatment weighting to account for baseline differences and to balance covariates, with standardized mean differences reported. Residual imbalance persisted in multiple covariates after inverse probability of treatment weighting (standardized mean difference >0.10), which was subsequently addressed by inclusion of these variables in the doubly adjusted Cox proportional hazard models. In addition, the current analysis was performed using the Society of Thoracic Surgeons/American College of Cardiology Transcatheter Valve Therapy registry, which contains missing data for multiple covariates, which Kumar et al addressed by multiple-imputation analysis. Consistent findings between the primary and the imputed analyses suggested that missing data were unlikely to significantly bias the study results.

A noteworthy limitation of this study is the arbitrary dichotomous BMI cutoff of 25 kg/m^2^ for the primary analysis. This approach differs significantly from previous literature, in which the analysis has been performed using the 4 traditional World Health Organization BMI categories of underweight, normal weight, overweight, and obese.^8^^,^^9^ Such investigator-defined arbitrary BMI categorization may obscure meaningful differences across BMI categories and introduce potential for misclassification bias. Although Kumar et al sought to address this by performing a sensitivity analysis by categorizing BMI into the 4 conventional categories, and noted findings similar to the primary analysis, the initial reliance of dichotomous BMI threshold limits interpretation and direct comparison to prior literature exploring the association between BMI and TAVR outcomes. Although obesity was associated with improved outcomes, especially with advancing age, excessive adiposity should not inherently be considered protective and benign. Rather, the association might be due to the health-deteriorating effect of undernutrition and frailty. Even though the authors adjusted for factors including left ventricular ejection fraction, the observed association may partly reflect reverse causality, whereby the patients with more advanced disease experience progressive weight loss and cardiac cachexia, resulting in poorer outcomes. In addition, it is well known that BMI is an improper marker of body composition and physiologic reserve.^10^ The inflection from focusing on obesity as a protective factor to nutrition, frailty, and sarcopenia being markers of vulnerability is an important perspective.

In summary, we congratulate the authors for a thoughtful and important contribution to the contemporary literature. Further prospective mechanistic studies are needed to elucidate the pathophysiologic pathways underlying the relationship between adiposity and outcomes following TAVR. More sophisticated pre-TAVR anthropometric measures, advanced imaging modalities, and incorporation of inflammatory markers may provide a more comprehensive characterization of physiologic reserve than BMI alone. Until such data are available, preprocedural frailty testing, nutrition assessment and counseling, and thorough risk stratification can help improve short-term procedural and long-term outcomes.

## Funding support and author disclosures

Dr Vallabhajosyula has served on the medical advisory board for Phillips and Chiesi; and has received consulting fees from for Abiomed/Johnson and Johnson and Marquet. All other authors have reported that they have no relationships relevant to the contents of this paper to disclose.
